# Delay and Speed of Visual Feedback of a Keystroke Cause Illusory Heaviness and Stiffness

**DOI:** 10.3389/fnins.2022.761697

**Published:** 2022-03-18

**Authors:** Takumi Yokosaka, Takahiro Kawabe

**Affiliations:** NTT Communication Science Laboratories, Nippon Telegraph and Telephone Corporation, Atsugi, Japan

**Keywords:** illusory heaviness, illusory stiffness, sense of agency, visual-tactile interaction, cross-modal integration

## Abstract

Imposing a delay between an action (e.g., a limb movement) and its related visual feedback (e.g., a cursor movement on the display) induces a peculiar sensation of heaviness or stiffness. Earlier studies have examined this delay-induced heaviness or stiffness sensation in relation to the non-arbitrary causal relationship between an action and its effect. Here, “non-arbitrary causal relationship” means that an action produces a specific and deterministic pattern of visual feedback; for example, a leftward limb movement consistently and deterministically causes a leftward visual motion. In modern graphical user interfaces, on the other hand, users often control visual information by pressing keys, wherein the relationship between the keystroke and the change in visual information is arbitrary. The present study examined whether the sensation of heaviness, stiffness and bumpiness could be caused when participants' keystroke produced a delayed arbitrary visual feedback. Participants were asked to press and hold down an assigned key to cause temporal luminance changes in a square centered on the display, an arbitrary visual feedback of their keystroke. Not only the onset delay of the temporal luminance change from the participant's keystroke but also the speed of the temporal luminance change were examined as a visual cue to heaviness, stiffness, or bumpiness. In Experiment 1, the participants' task was to give a rating for the strength of the heaviness, stiffness, or bumpiness perceived when they pressed the key. Our results showed that the heaviness and stiffness ratings increased as the delay increased and decreased as the speed increased. To check whether the manipulation of the delay and speed of the visual feedback caused changes in the subjective evaluation of sensorimotor incongruence, in Experiment 2, we asked the participants to give a rating for the sense of agency. The rating scores decreased as the delay increased and increased as the speed increased. The delay and speed influenced the rating scores for the sense of agency in the opposite direction to those for heaviness/stiffness. We discuss that the brain determines the heaviness and stiffness during a keystroke based on internalized statistics relating to the delay and speed of the action feedback.

## 1. Introduction

In various human-machine interfaces, we perceive a causal relationship between our actions and the sensory feedback they trigger. For example, when a keystroke causes a change in the visual display, we may feel as if we were the one who caused the change. This sort of feeling is called a “sense of agency” (Gallagher and Gallagher, 2000; Farrer et al., 2003; Jeannerod, 2003).

Stimulus parameters to determine the sense of agency have been examined in perceptual and cognitive studies. In particular, a delay between an action and its effect is a contributing factor to a deterioration in the sense of agency (Shanks et al., 1989; Sato and Yasuda, 2005; Asai and Tanno, 2007; Farrer et al., 2013; Kawabe, 2013; Kawabe et al., 2013; Wen et al., 2015a,b). There are several explanations for the effect of delayed feedback on the sense of agency. First, the majority of studies assume that the brain has a comparator for predicted and actual sensory feedback of an action. When the comparator detects a delay between an action and its feedback, the brain interprets that the feedback is not triggered by the action and tries to calibrate the prediction system for future feedback (Blakemore et al., 1998, 1999; Haggard et al., 2002; Chambon et al., 2014; Swiney and Sousa, 2014 but also read Synofzik et al., 2008). Another line of study tries to explain the sense of agency in terms of cross-modal grouping. Specifically, with a large temporal separation between a participant's own keystroke and the feedback it triggers, cross-modal perceptual grouping (or cross-modal perceptual correspondence between them as suggested by Nishida and Johnston, 2002; Fujisaki and Nishida, 2007) is impaired and this perhaps reduces the sense of agency (Kawabe et al., 2013).

A delay between an action and the visual feedback of her/his body parts also induces sensations of heaviness and stiffness in relation to the feedback. For example, using an experimental setting in which a hand position was displayed as a cursor, Honda et al. (2013) showed that a larger delay of the cursor feedback could produce a greater sensation of cursor heaviness. When adding a delay to visual feedback of a participant's hand image while she/he periodically moved their hand, Osumi et al. (2018) observed that the participant reported that her/his limb became perceptually heavier as the visual feedback delay was increased. Using the mixed reality setting, Di Luca et al. (2011) investigated how force and/or visual delays altered the perception of object compliance and found that a visual delay led to a lower perceived sense of compliance, while a force delay led to a higher perceived sense of compliance. Kambara et al. (2013) also showed a similar relationship between force/visual delay and weight perception.

The detection of sensorimotor congruence is one of the key components of the heaviness and stiffness sensation. According to Honda et al. (2013), sensorimotor incongruence due to delayed visual feedback may lead to a dissociation between predicted and actual feedback of action, which may trigger the illusory sense of heaviness (and stiffness). Currently, it is unclear what kind of mechanisms mediate the detection of sensorimotor incongruence. One candidate is brain processing in the cerebellum. Blakemore and Sirigu (2003) showed that activation in the right cerebellar hemisphere increased with the temporal discrepancy between a participant's action and its sensory consequence, which indicates that the cerebellum is one of the neural substrates responsible for the prediction of the sensory outcome of a participant's action. The other candidate is a cognitive or perceptual factor that possibly operates independently of sensorimotor processing (Flanagan and Beltzner, 2000). In either case, the illusory heaviness and stiffness possibly come from the re-selection of the internal model due to sensorimotor incongruence (Takamuku and Gomi, 2015). Illusory heaviness and stiffness of this sort has been investigated as a pseudo-haptics technique in engineering contexts (Dominjon et al., 2005; Argelaguet et al., 2013; Punpongsanon et al., 2015; Samad et al., 2019).

As regards illusory heaviness and stiffness, there are two important questions that need to be addressed. The first question is whether the illusory heaviness and stiffness can be produced with the visual factors related to a keystroke. So far, previous studies (Di Luca et al., 2011; Honda et al., 2013; Osumi et al., 2018) have examined illusory heaviness and stiffness using a non-arbitrary causal relationship between an action and its feedback. Here, “non-arbitrary causal relationship” means that a participant's action produces a specific and deterministic pattern of visual feedback; for example, a leftward limb movement consistently and deterministically causes a leftward visual motion. Hence, it was still unclear whether a delay in visual feedback could function to produce the illusory heaviness and stiffness even when the feedback was triggered by a keystroke in such as way that it did not have a non-arbitrary causal relationship with the feedback. Frey et al. (2015) showed that an arbitrary causal relationship between a button press and subsequent robotic arm movements activated the right cerebellar hemisphere, which likely mediated the prediction of forthcoming sensory signals after the button press. That is, without a non-arbitrary causal relationship between an action and its outcome, the cerebellum seems to predict the timing of arbitrary feedback of a keystroke. Indeed, the cerebellum also mediates the perceptual prediction of external events (OReilly et al., 2008). Since the re-selection of the internal model due to sensorimotor incongruence possibly underlies the illusory heaviness and stiffness (Takamuku and Gomi, 2015), there is a possibility that the temporal dissociation between a keystroke and the delayed visual feedback it triggers can be interpreted as stemming from physical events relevant to heaviness and stiffness even when the keystroke is related to the visual feedback only in an arbitrary manner (i.e., without a spatiotemporally-correlated relationship between the action and its outcome).

Speed is another factor in visual feedback. Previous studies on material perception have shown that image speed and/or playback speed of video clips were strong determinants of apparent heaviness and stiffness (Shim et al., 2009; Kawabe et al., 2015; Kawabe and Nishida, 2016; Bi et al., 2018). In visual stimuli inducing perceptual causality (Michotte, 1963; Scholl and Tremoulet, 2000; Meding et al., 2020; Wang et al., 2020), movement speeds after object collision determine the perceived heaviness of the object (Todd and Warren, 1982). A previous study (Kawabe et al., 2021) showed that a lower speed of a cursor controlled by a participant's keystroke caused a stronger sense of resistance, while the resistance sensation produced by visual processing is not necessarily linked to the participant's action.

The second question is whether illusory heaviness and stiffness are related to the sense of agency. As described above, Honda et al. (2013) reported that the illusory heaviness for the delayed feedback of a participant's hand position was possibly due to a prediction error for the feedback. It is also well known that the sense of agency often deteriorates when the prediction error for an action feedback is large (Haggard et al., 2002; Chambon et al., 2014; Swiney and Sousa, 2014). In these respects, we predicted that the illusory heaviness and stiffness would be negatively correlated with the sense of agency. Specifically, the rating scores for the illusory heaviness and stiffness would increase as the delay in visual feedback increased, while the rating scores for the sense of agency would decrease as the delay in visual feedback increased.

To answer these questions, we conducted the first experiment to examine whether illusory heaviness and stiffness could be induced by the visual feedback made by a participant's keystroke, manipulating the delay of visual feedback onsets as well as the speed of visual feedback changes. To check the relationship between illusory heaviness/stiffness and sense of agency, in the second experiment we tested how the two stimulus parameters modulated the sense of agency.

The present study used temporal luminance changes as visual stimuli. Temporal luminance changes have been found to stimulate the motion processing of the human visual system (Anstis, 1967, 1990; Arnold and Anstis, 1993), and thus, can be used to examine the effect of visual speed on illusory heaviness and stiffness. Because temporal luminance changes are not involved with spatial stimulus shift, the undesired effect of attentional shifts and eye movements can be reduced. Moreover, temporal luminance changes are not easily related to the spatiotemporal pattern of a participant's hand movement, and thus are useful to investigate how an arbitrary causal relationship between a participant's keystroke and its feedback is related to the induction of illusory heaviness and stiffness.

## 2. Experiment 1

### 2.1. Purpose

The purpose of the experiment was to examine whether the participants could feel heaviness and stiffness in relation to a temporal luminance change triggered by their keystrokes. In the experiment, the participants were asked to press and hold down a key on a keyboard to initiate a temporal luminance change on a display. The delay of the onset of the temporal luminance changes from the timing of the participant's keystroke was varied between five different levels: 0, 250, 500, 750, and 1,000 ms. Also, the speed of the temporal luminance change was varied by modulating the duration of the change itself between three different levels. The effects of delay and speed on the degree of perceived heaviness and stiffness were tested.

In addition, we compared the effects on the evaluations of heaviness and stiffness to those on the evaluation of bumpiness to check the possibility that any evaluation could be affected by the delay and speed in the same way as that for the heaviness and stiffness. Earlier studies (Lcuyer et al., 2008; Mensvoort et al., 2008, 2010; Costes et al., 2019) showed that the sensation of bumpiness could be generated by systematically manipulating the magnitude of displacement and size of a mouse cursor along its trajectory. Changes in the appearance of the mouse cursor may stimulate visual functions to perceive structure from motion (Andersen and Bradley, 1998) and thus can be used as a cue to visual depth perception. On the other hand, because the onset delay of the uniform temporal change does not contribute to the perception of structure from motion, it was expected that the delay would not affect the evaluation of bumpiness. On the other hand, it was unclear whether the speed of the temporal luminance change could influence the evaluation of bumpiness. In this respect, the effect of the speed on the bumpiness perception was an exploratory component of this experiment.

### 2.2. Method

#### 2.2.1. Participants

Three hundred and sixty-two people participated in the experiment. Participants were divided into three groups, each performing one of three tasks: heaviness evaluation, stiffness evaluation, or bumpiness evaluation. The reason for assigning different participants to different tasks was to avoid unintended effects, such as a participants evaluation of one item simultaneously affecting that of other items. One hundred and twenty-two participants (61 females) participated in the heaviness evaluation task and the mean ± standard deviation (SD) of their age was 40.29 ± 11.52. One hundred and twenty participants (60 females) participated in the stiffness evaluation task and the mean ± SD of their age was 39.66 ± 11.07. One hundred and twenty participants (59 females) participated in the bumpiness evaluation task and the mean ± SD of their age was 40.03 ± 11.68. A Japanese crowdsourcing research company recruited the participants online and they were paid for their participation. The participants were recruited through invitation messages they would have received. In the message, they were informed, “You can earn tens or hundreds of yen worth of points as a reward for a 10-min participation in this experiment.” They were unaware of the specific purpose of the experiment. Ethical approval for this study was obtained from the ethics committee at Nippon Telegraph and Telephone Corporation (Approval number: R02-009 by NTT Communication Science Laboratories Ethics Committee). The experiments were conducted according to principles that have their origin in the Helsinki Declaration. Written informed consent was obtained from all observers in this study.

#### 2.2.2. Apparatus

The experiment conducted in this study was carried out using the participants' own personal computers (PC) because our experimental script could only be run on a PC. Hence, smartphones or tablet PCs, which do not have keyboards, could not be used in this experiment. Viewing distance and screen size were not controlled because their effect was not evident in the preliminary observation provided the user used the PC normally.

#### 2.2.3. Stimuli

**Figure 1:**
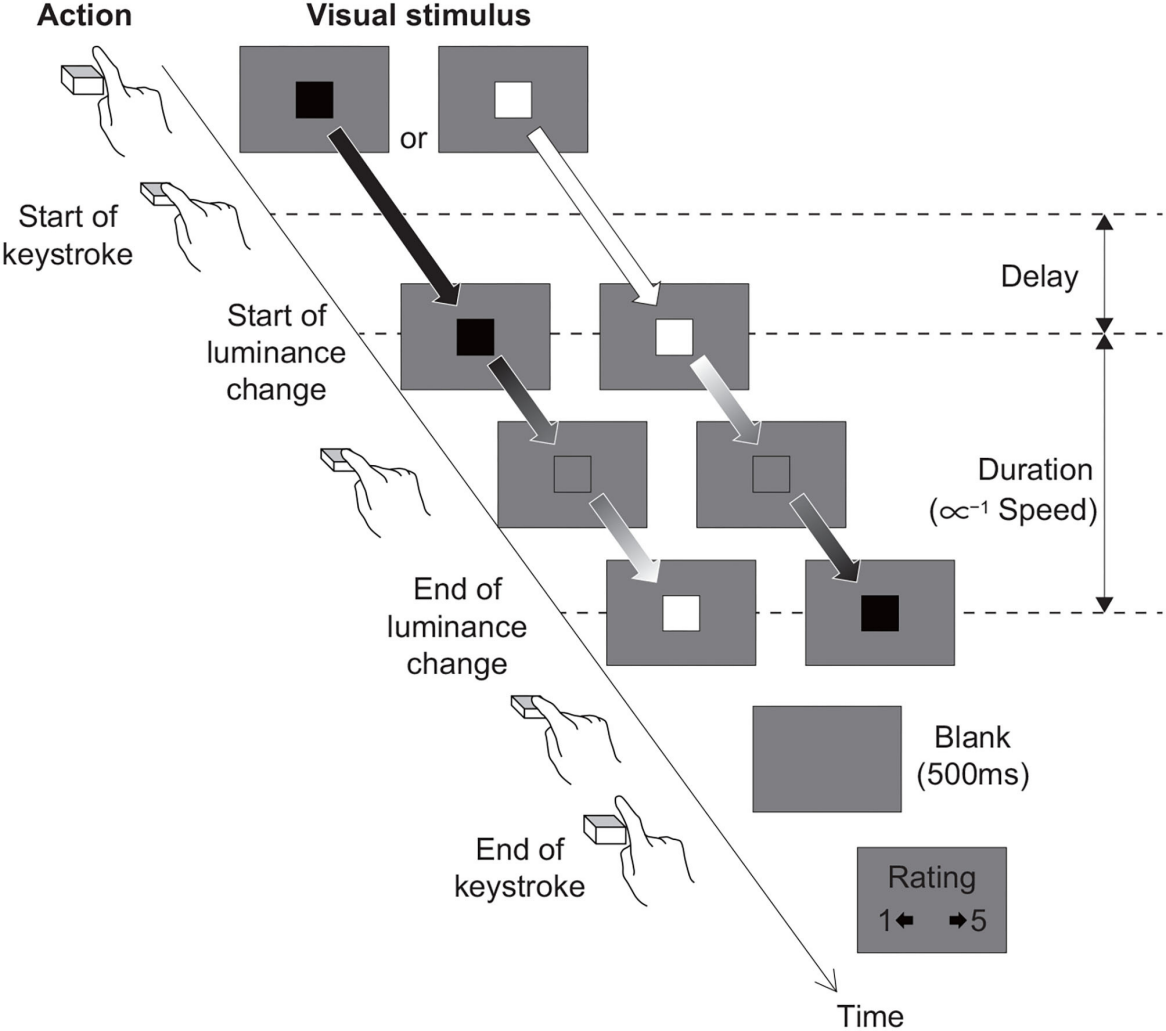
Trial sequence of experiments.

As shown in Figure 1, the stimuli consisted of a square [100 × 100 pixels, with RGB values of (0, 0, 0) or (255, 255, 255)] centered in the display and a uniform background [with RGB values of (128,128,128)]. The initial luminance of the square in RGB values was randomly set to (0, 0, 0) or (255, 255, 255) and changed toward (255, 255, 255) or (0, 0, 0), respectively, only when the participant held down an assigned key (i.e., the M key on the computer keyboard). When participants released the key before the luminance of the square had finished changing, the luminance of the square returned to its initial state and the message “The ‘M' key was released in the middle (of the stimulus presentation)” was displayed. Note that the “of the stimulus presentation” is a supplement to the translation into English, and the meaning is understood in Japanese even without it. The square took 500, 1,000, or 2,000 ms to complete the luminance change: the speed at which the luminance changed was (510.0, 510.0, 510.0)/s (fast), (255.0, 255.0, 255.0)/s (medium), or (127.5, 127.5, 127.5)/s (slow), respectively. The conditions relevant to the speed of luminance change are referred to as “speed conditions.” Also, there was a programmed delay of 0, 250, 500, 750, or 1,000 ms for the luminance of the square to start changing after the participant pressed the key. The conditions relevant to these time lags are referred to as “delay conditions.” We rendered the luminance of the square based on the time elapsed from the start of the key press, not on a frame rate basis. Therefore, the delay and speed were not substantially affected by the frame rate of a participant's PC. We preliminarily measured rough latency, i.e., how long it took for the luminance of the square to change after pressing a key in the author's computer. The latency likely stemmed from system delay and the mean ± SD of the latency for the three measurements was 76.12 ± 8.02 ms. Moreover, the latency did not depend on the delay and speed conditions (see Section S2 in Supplementary Material). After the square had completed the luminance change, it disappeared for 500 ms and then the answer screen was displayed.

#### 2.2.4. Procedure

At the beginning of the experiment, the participants were presented with written instructions that described the situation and their tasks in the experiment. After reading this, the participant pressed the space key on the computer keyboard to start the experiment. The participant's task was to hold down the M key and trigger the luminance change of the square. After the square had completed the luminance change and disappeared, the answer screen was displayed. In the heaviness, stiffness, or bumpiness evaluation tasks, the following instruction sentences were presented to the participants, “Evaluate the sense of heaviness you felt while controlling the square's luminance change,” “Evaluate the sense of stiffness you felt while controlling the square's luminance change,” or “Evaluate the sense of bumpiness you felt while controlling the square's luminance change,” respectively. In the instruction sentences, we did not included any terms implying either haptic (e.g., muscle effort and key resistance) or visual (e.g., the square seems heavy) aspects of the heaviness/stiffness sensation because we believe that the heaviness/stiffness sensation is a product of bi-modal processing or cross-modal integration of the participants self-action and its outcome, and hence, it is not appropriate to have the participants focus on either haptics or vision. In all tasks, participants reported the evaluation on a 5-point scale by pressing the assigned keys, wherein 5 represented the highest heaviness, stiffness, or bumpiness, while 1 represented the lowest heaviness, stiffness, or bumpiness. After reporting the evaluation, the next trial began. Each experimental condition was repeated 5 times. Thus, each participant performed 75 trials (3 speed conditions × 5 delay conditions × 5 iterations). We randomized the order of presentation every 15 trials (i.e., 3 speed conditions × 5 delay conditions). Moreover, we measured effective frames per second (efps) and calculated the averaged efps during stimulus presentation on each trial to check whether the participant's PC was not able to draw the experimental stimuli accurately due to the efps being too low.

### 2.3. Results and Discussion

The averages (SD) of mean efps among participants PCs in the heaviness, stiffness, and bumpiness evaluation tasks were 58.38 (6.69) Hz, 57.89 (7.70) Hz, and 58.29 (6.88) Hz, respectively (Supplementary Figure 12A). The averages of SD of efps among participants' PCs in the heaviness, stiffness, and bumpiness evaluation tasks were 0.33, 0.32, and 0.34 Hz, respectively (Supplementary Figure 12B). To see if the PC of a participant whose results showed a small mean value of efps also showed a large standard deviation in efps, we plotted the relationship between the mean and standard deviation of efps for each participant (Supplementary Figure 12C). This result shows that participants' PCs with smaller mean efps showed smaller standard deviations of efps. The results showed that for most participants the experiments were performed at almost 60 efps with small fluctuations, which suggests that it is unlikely that the rendering performance of participants' PCs differed significantly between trials.

**Figure 2:**
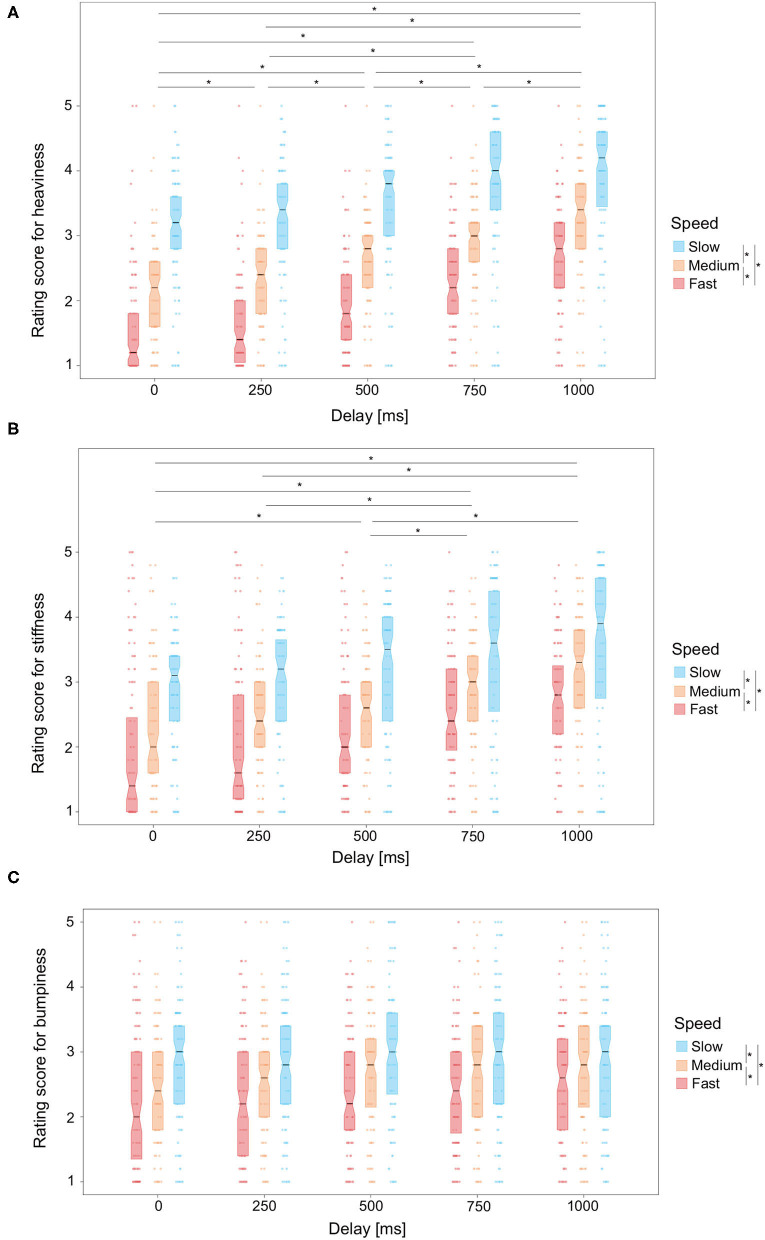
Experiment 1 results. Box plots of **(A)** heaviness rating scores, **(B)** stiffness rating scores, and **(C)** bumpiness rating scores for each of the speed conditions as a function of the delay. Asterisks denote the pairs for which the means were significantly different using the EcolUtils package (Bonferroni-corrected *p* < 0.05).

For each condition, heaviness rating scores were averaged for each participant. The mean and variance values are shown in Table 1. Rating scores across the participants are shown in Figure 2A. By using “vegan” (Oksanen et al., 2020) and “EcolUtils” (Salazar, 2020) packages of R (Team, 2020), a two-way permutation analysis of variance (Anderson, 2001; Anderson and Walsh, 2013) was conducted with the speed and delay conditions as within-subject factors. The permutation analysis of variance is a non-parametric multivariate statistical test and does not require any assumptions about the data distribution. The main effect of the delay condition was significant (*F* = 74.24, *p* < 0.001, *r*^2^ = 0.10). The main effect of the speed condition was also significant (*F* = 371.32, *p* < 0.001, *r*^2^ = 0.25). The interaction between them was significant (*F* = 9.93, *p* < 0.001, *r*^2^ = 0.03). The simple main effect of delay was also significant for all speed conditions (Table 2). The simple main effect of speed was significant for all delay conditions (Table 2).

**Table 1:** Average (standard deviation) of heaviness rating scores.

	**Delay = 0 [ms]**	**250 [ms]**	**500 [ms]**	**750 [ms]**	**1,000 [ms]**
Speed = slow	3.10 (0.88)	3.24 (0.94)	3.49 (0.94)	3.76 (1.04)	3.85 (1.04 )
Speed = medium	2.13 (0.72)	2.32 (0.70)	2.61 (0.72)	2.89 (0.73)	3.22 (0.88 )
Speed = fast	1.51 (0.78)	1.65 (0.74)	1.92 (0.73)	2.32 (0.77)	2.67 (0.79 )

**Table 2:** Simple main effects of delay and speed on heaviness evaluation.

	***F***	**Bonferroni corrected *p***	***r* ^2^**
Speed = slow	7.87	< 0.001	0.05
Speed = medium	30.05	< 0.001	0.17
Speed = fast	55.56	< 0.001	0.27
Delay = 0 [ms]	120.98	< 0.001	0.40
Delay = 250 [ms]	106.58	< 0.001	0.37
Delay = 500 [ms]	87.76	< 0.001	0.33
Delay = 750 [ms]	58.40	< 0.001	0.24
Delay = 1,000 [ms]	31.99	< 0.001	0.15

In a way similar to the heaviness rating scores, the stiffness rating scores were averaged for each participant. The mean and variance values are shown in Table 3. We conducted a two-way permutation analysis of variance for the stiffness rating scores (Figure 2B). Both of the main effects of the delay condition (*F* = 28.09, *p* < 0.001, *r*^2^ = 0.05) and the speed condition (*F* = 87.90, *p* < 0.001, *r*^2^ = 0.08) were significant. The interaction between them was also significant (*F* = 4.66, *p* < 0.001, *r*^2^ = 0.02). The simple main effect of delay was also significant for all speed conditions (Table 4). The simple main effect of speed was significant for all delay conditions (Table 4).

**Table 3:** Average (standard deviation) of stiffness rating scores.

	**Delay = 0 [ms]**	**250 [ms]**	**500 [ms]**	**750 [ms]**	**1,000 [ms]**
Speed = slow	2.86 (0.91)	2.96 (0.95)	3.15 (1.07)	3.32 (1.23)	3.47 (1.33)
Speed = medium	2.29 (0.94)	2.46 (0.83)	2.62 (0.79)	2.85 (0.84)	3.11 (0.97)
Speed = fast	1.94 (1.17)	2.06 (1.11)	2.23 (1.00)	2.54 (0.90)	2.78 (0.86)

**Table 4:** Simple main effects of delay and speed on stiffness evaluation.

	***F***	**Bonferroni corrected *p***	***r* ^2^**
Speed = slow	3.06	0.04	0.02
Speed = medium	13.32	< 0.001	0.08
Speed = fast	21.47	< 0.001	0.13
Delay = 0 [ms]	32.94	< 0.001	0.15
Delay = 250 [ms]	29.26	< 0.001	0.14
Delay = 500 [ms]	23.94	< 0.001	0.12
Delay = 750 [ms]	11.60	< 0.001	0.06
Delay = 1,000 [ms]	7.26	< 0.001	0.04

The bumpiness rating scores were also averaged for each participant. The mean and variance values are shown in Table 5. Using the bumpiness rating scores, we conducted a two-way permutation analysis of variance for them (Figure 2C). The main effect of the speed condition was significant (*F* = 34.93, *p* < 0.001, *r*^2^ = 0.04) while that of the delay condition was not significant (*F* = 2.28, *p* = 0.054, *r*^2^ < 0.01). The interaction between them was also not significant (*F* = 0.92, *p* = 0.51, *r*^2^ < 0.01).

**Table 5:** Average (standard deviation) of bumpiness rating scores.

	**Delay = 0 [ms]**	**250 [ms]**	**500 [ms]**	**750 [ms]**	**1,000 [ms]**
Speed = slow	2.85 (0.93)	2.83 (0.95)	2.97 (1.01)	2.88 (1.02)	2.87 (1.01)
Speed = medium	2.49 (0.86)	2.56 (0.84)	2.70 (0.91)	2.71 (0.93)	2.70 (0.94)
Speed = fast	2.26 (1.06)	2.32 (0.96)	2.41 (0.94)	2.44 (0.88)	2.54 (0.91)

The results showed that the rating scores for heaviness and stiffness increased with the delay between the timing of a participant's keystroke and the onset of a temporal luminance change, whereas the rating scores for bumpiness did not. This is the first evidence that humans can estimate heaviness and stiffness from a delay in visual feedback that are caused by a participant's keystroke. The results are consistent with previous studies suggesting that the brain perceptually estimates heaviness and stiffness by re-selecting the internal model (Takamuku and Gomi, 2015) or by interpreting prediction errors (Honda et al., 2013) on the sensorimotor incongruence in terms of timing between a participant's keystroke and the feedback it triggers. In contrast, the results showing that the perception of bumpiness was not influenced by the delay in visual feedback indicate that bumpiness is not grounded in the computation of sensorimotor incongruence. The brain possibly uses different information for the estimation of bumpiness from that used for the estimation of heaviness and stiffness.

Consistent with previous studies (Todd and Warren, 1982; Shim et al., 2009; Kawabe et al., 2015; Kawabe and Nishida, 2016; Bi et al., 2018) on material perception, the speed of the feedback transition also influenced the heaviness and stiffness ratings. Specifically, the slower temporal luminance changes caused a greater perception of heaviness and stiffness. The results indicate that the illusory heaviness and stiffness on the basis of the delayed visual feedback of a participant's keystroke stem from both the re-selection of the internal model due to sensorimotor incongruence and the appearance of the feedback itself. Unexpectedly, the perception of bumpiness was influenced by the speed of temporal luminance changes. One possible explanation for the speed effect might be that it is evidence for certain demand characteristics or response bias since the speed parameter is affected in the same way regardless of the participants evaluations. Moreover, the range of rating score variations is rather smaller for the bumpiness than for the heaviness and stiffness ratings, and thus, the speed of the temporal luminance changes perhaps played only a minor role in determining the bumpiness rating. Rather, we would like to emphasize the results showing that the perception of bumpiness was not influenced by the delay applied to the visual feedback, which indicates that the effect of the delay on the heaviness and stiffness ratings is not based on the demand characteristics.

**Figure 3:**
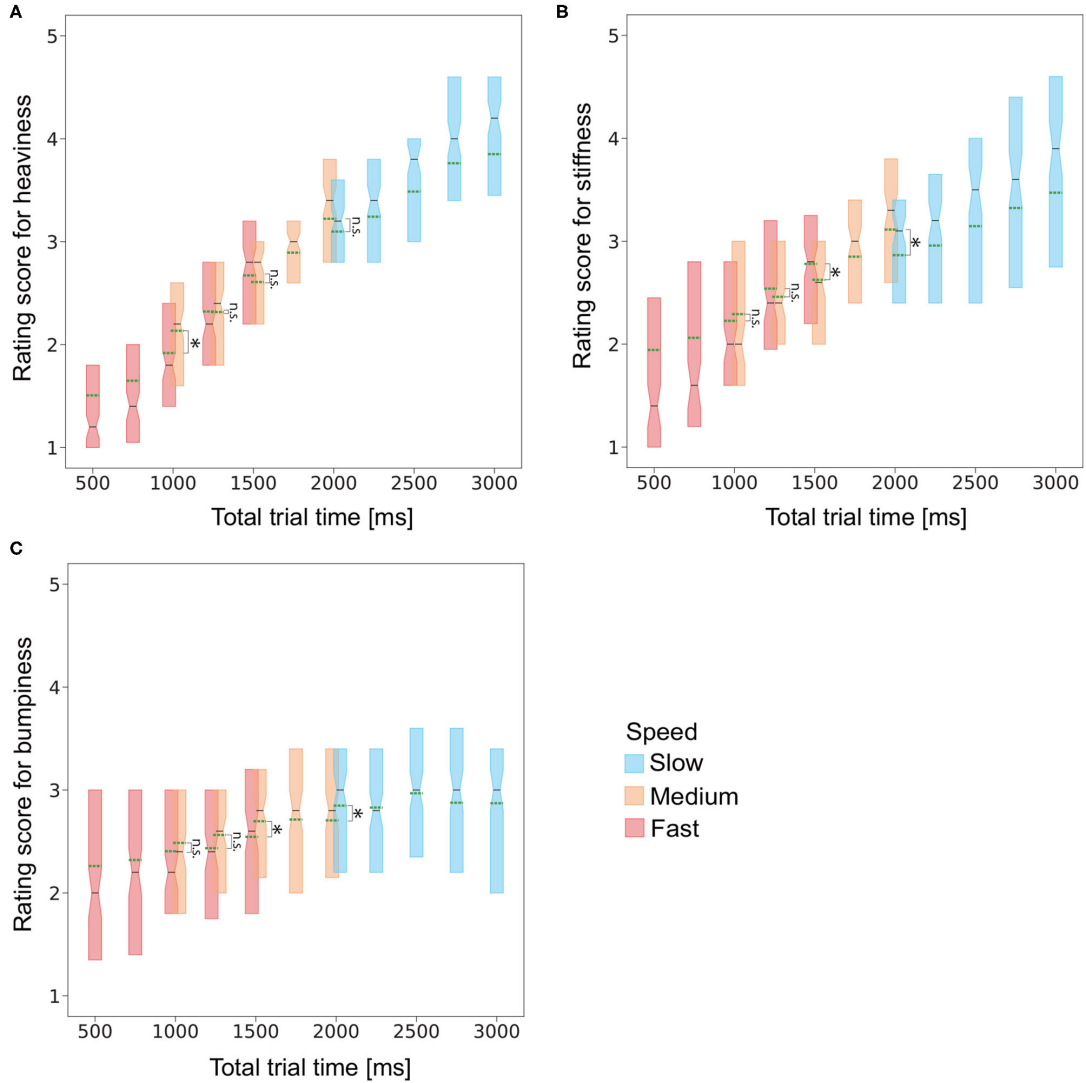
Box plot of the **(A)** heaviness, **(B)** stiffness, and **(C)** bumpiness rating scores as a function of total trial time. Green dashed lines denote the averaged values of the rating scores. The Asterisks and n.s. denotes that the difference was significant (i.e., Bonferroni corrected *p* < 0.05) and not significant (i.e., Bonferroni corrected *p* > 0.05), respectively.

One might suspect that the results could have been explained by a single parameter, total trial time (i.e., duration of luminance change + delay), rather than by the combination of the two independent parameters, delay and speed (i.e., duration; note that the durations under the fast, medium, and slow speed conditions were 500, 1,000, and 2,000 ms, respectively). Plots of the sense of heaviness, stiffness, and bumpiness rating scores as a function of total trial time (Figure 3) indeed show a linear relationship between them. Regression analyses of rating scores using speed, delay, combinations of speed and delay, and total trial time showed that total trial time could explain a large part of the rating scores (Table 6). Here, to exploratively investigate whether the same total trial time gives the same rating scores regardless of each value of delay and duration (i.e., speed), we compared pairs with a total trial time of 1,000 ms [i.e., (duration = 500 and delay = 500) vs. (duration = 1,000 and delay = 0)], 1,250 ms [i.e., (duration = 500 and delay = 750) vs. (duration = 1,000 and delay = 250)], 1,500 ms [i.e., (duration = 500 and delay = 1,000) vs. (duration = 1,000 and delay = 500)], and 2,000 ms [i.e., (duration = 1,000 and delay = 1,000) vs. (duration = 2,000 and delay = 0)]. We employed the bootstrap method (Efron and Tibshirani, 1994) to assess whether the averaged rating scores of heaviness, stiffness, and bumpiness for the pairs were different or not. We found that the averaged heaviness rating score under the condition (duration = 1,000 ms and delay = 0 ms) was significantly higher than that under the condition (duration = 500 ms and delay = 500 ms) (Cliff's delta = 0.21, Bonferroni corrected *p* < 0.001) and the averaged heaviness rating score under the condition (duration = 1,000 ms and delay = 1,000 ms) was significantly higher than that under the condition (duration = 2,000 ms and delay = 0 ms) (Cliff's delta = 0.10, Bonferroni corrected *p* < 0.001). The averaged stiffness rating score under the condition (duration = 500 ms and delay = 1,000 ms) was significantly higher than that under the condition (duration = 1,000 ms and delay = 500 ms) (Cliff's delta = 0.12, Bonferroni corrected *p* < 0.05) and the average score under the condition (duration = 1,000 ms and delay = 1,000 ms) was significantly higher than that under the condition (duration = 2,000 ms and delay = 0 ms) (Cliff's delta = 0.17, Bonferroni corrected *p* < 0.001). The averaged bumpiness rating score under the condition (duration = 1,000 ms and delay = 500 ms) was significantly higher than that under the condition (duration = 500 ms and delay = 1,000 ms) (Cliff's delta = 0.09, Bonferroni corrected *p* < 0.05) and the average score under the condition (duration = 2,000 ms and delay = 0 ms) was significantly higher than that under the condition (duration = 1,000 ms and delay = 1,000 ms) (Cliff's delta = 0.09, Bonferroni corrected *p* < 0.05). The average scores were not significantly different between the other pairs. These results suggest that the total trial time is possibly a major parameter for explaining the rating scores, while the two independent parameters, speed and delay, also somehow impact independently on the scores. Based on the major role of the total trial time, the effect of speed can be interpreted as the effect of stimulus duration. To further disentangle the contributions of the total trial time, speed and stimulus duration, it is necessary to test conditions wherein these parameters are varied independently in future studies.

**Table 6:** Adjusted coefficients of determination for each model.

	**Speed + Delay**	Speed * Delay	**Total trial time**	**Speed**	**Delay**
Heaviness	0.98	0.98	0.98	0.69	0.21
Stiffness	0.98	0.98	0.95	0.57	0.33
Bumpiness	0.90	0.93	0.87	0.81	0.02
Sense of agency	0.98	0.98	0.94	0.52	0.38
Background luminance	0.94	0.93	0.74	0.94	−0.07

## 3. Experiment 2

### 3.1. Purpose

This experiment examined how the illusory heaviness and stiffness could be related to the subjective measure of the sensorimotor incongruence, sense of agency. As described above, re-selection of the internal model (Takamuku and Gomi, 2015) or interpretation of prediction errors (Honda et al., 2013) on the sensorimotor incongruence possibly underlies the illusory heaviness and stiffness. The sensorimotor incongruence undermines the sense of agency (Shanks et al., 1989; Sato and Yasuda, 2005; Asai and Tanno, 2007; Farrer et al., 2013; Kawabe, 2013; Kawabe et al., 2013; Wen et al., 2015a,b). Taken together, a larger sensorimotor incongruence likely causes a stronger sensation of heaviness and stiffness while also causing a weaker sense of agency. Based on this idea, it was predicted that a larger onset delay of the temporal luminance changes would cause lower rating scores for the sense of agency. It was also predicted that a lower speed of the temporal luminance changes would cause the rating scores for the sense of agency since it was known that the speed of visual feedback affects the sense of agency (Kawabe, 2013). Moreover, we also expected that the rating scores for the sense of agency in this experiment would be influenced by the delay and speed in the opposite direction to the rating scores for the heaviness and stiffness. In a control condition to check whether the effect of the delay on the sense of agency (SoA) ratings stemmed from demanded characteristics, we also asked another group of participants to evaluate a background luminance change that was related neither to a participants key press nor to temporal luminance changes in the square.

### 3.2. Method

#### 3.2.1. Participants

Two hundred and thirty-nine people, who had not participated in Experiment 1, participated in Experiment 2. Participants were divided into two groups, each performing either of two tasks: SoA evaluation or background-luminance evaluation (i.e., the control question). One hundred and twenty participants (60 females) participated in the SoA evaluation task and the mean ± SD of their age was 39.40 ± 11.18. One hundred and nineteen participants (59 females) participated in the background-luminance evaluation task and the mean ± SD of their age was 40.03 ± 11.18.

#### 3.2.2. Stimuli

The stimuli in the SoA evaluation task were identical to those used in Experiment 1. On the other hand, the background-luminance evaluation task featured an additional five catch trials. In the catch trials, the stimuli and time course were identical to those used in the SoA evaluation tasks except for the following: the initial luminance of the background with RGB values (128, 128, 128) changed toward (0, 0, 0) or (255, 255, 255) with a 0 ms delay only when the participant held down an assigned key (i.e., the “M” key on the computer keyboard).

#### 3.2.3. Procedure

The procedure was identical to that used in Experiment 1 except for the following. In the SoA evaluation task, the following instruction sentence was given to the participants, “Rate the extent to which your key press appeared to causally control the luminance of the square.” They reported the evaluation on a 5-point scale by pressing the assigned keys, where 5 indicated the strongest impression and 1 the weakest impression of causal control. In the SoA evaluation task, each participant performed 75 trials [(3 speed conditions × 5 delay conditions) × 5 iterations]. We randomized the order of presentation every 15 trials (i.e., 3 speed conditions × 5 delay conditions) among participants. In the background-luminance evaluation task, the following instruction sentence was given to the participants, “Rate the degree to which the luminance of the background (not the square) changed.” They reported the evaluation on a 5-point scale by pressing the assigned keys, where 5 indicated the strongest impression and 1 the weakest impression of background luminance change. In the background-luminance evaluation task, each participant performed 80 trials [(3 speed conditions × 5 delay conditions + 1 catch trial) × 5 iterations]. We randomized the order of presentation every 16 trials (i.e., 3 speed conditions × 5 delay conditions + 1 catch trial) among participants.

### 3.3. Results and Discussion

The averages (SD) of efps among participants' PCs in the SoA evaluation and background-luminance evaluation tasks were 58.44 (7.73) Hz and 58.54 (6.26) Hz, respectively (see two plots on the right side of Supplementary Figure 12A). The averages of SD of efps among participants' PCs in the SoA evaluation and background-luminance evaluation tasks were 0.31 and 0.41 Hz, respectively (see two plots on the right side of Supplementary Figure 12B). The results showed that most participants' PC performed the experiments at almost 60 efps with small fluctuations, which suggests that it is unlikely that the rendering performance of participants' PCs differed significantly between trials.

**Figure 4:**
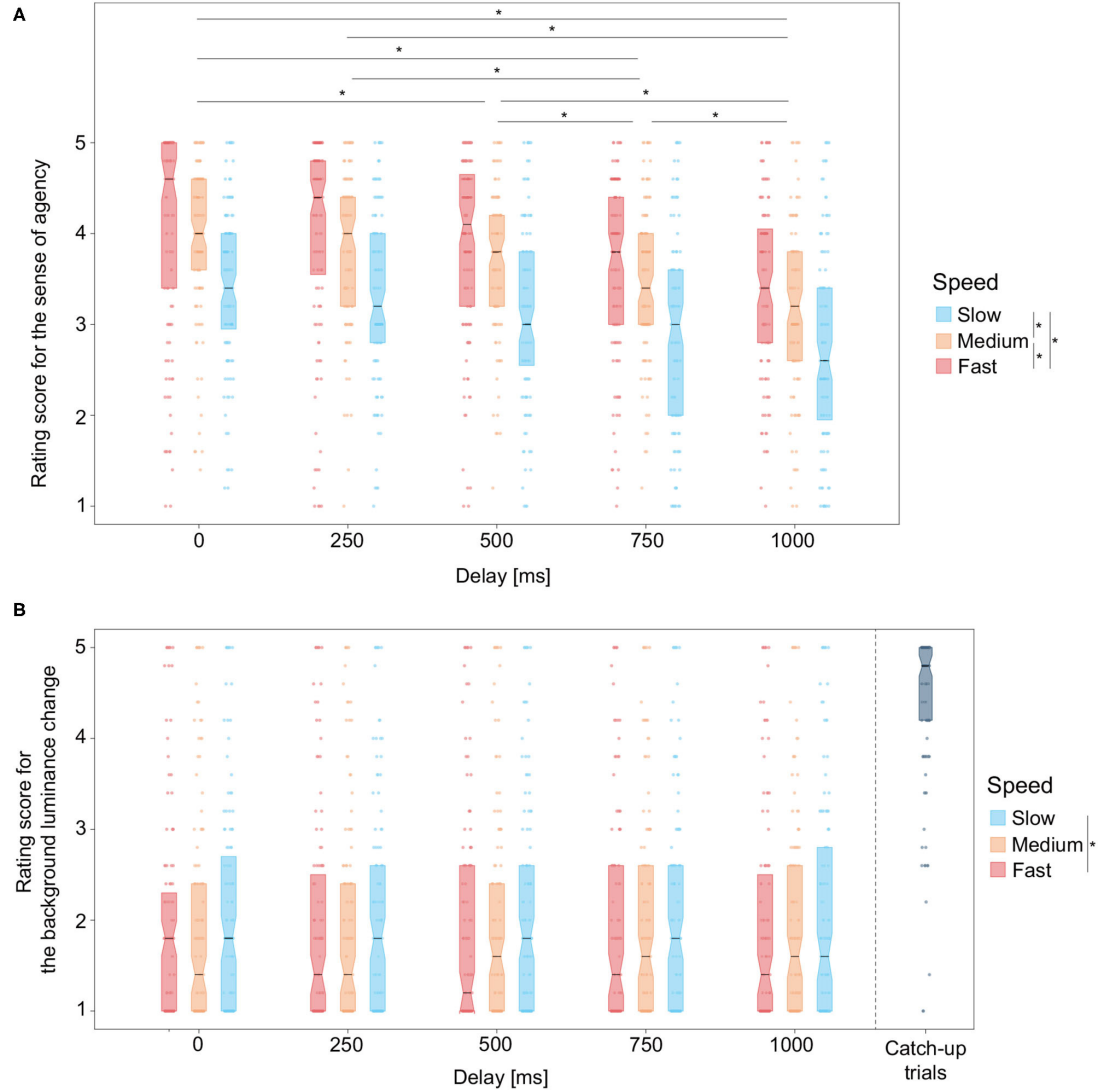
Experiment 2 results. Box plots of **(A)** sense of agency rating scores and **(B)** background-luminance rating scores for each of the speed conditions as a function of the delay. Asterisks denote the pairs for which the means were significantly different using the EcolUtils package (Bonferroni-corrected *p* < 0.05).

For each condition, SoA ratings scores were averaged for each participant. The mean and variance values are shown in Table 7. Rating scores across the participants are shown in Figure 4A. Using the individual mean rating scores, a two-way permutation analysis of variance was conducted with the speed and delay conditions as within-subject factors. The main effect of the delay condition was significant (*F* = 25.63, *p* < 0.001, *r*^2^ = 0.05). The main effect of the speed condition was also significant (*F* = 69.54, *p* < 0.001, *r*^2^ = 0.07). The interaction between them was not significant (*F* = 1.50, *p* = 0.135, *r*^2^ < 0.01).

**Table 7:** Average (standard deviation) of sense of agency rating scores.

	**Delay = 0 [ms]**	**250 [ms]**	**500 [ms]**	**750 [ms]**	**1,000 [ms]**
Speed = slow	3.44 (0.88)	3.33 (0.94)	3.1 (0.95)	2.93 (1.09)	2.72 (1.07)
Speed = medium	3.96 (0.82)	3.82 (0.81)	3.69 (0.76)	3.49 (0.83)	3.17 (0.89)
Speed = fast	4.09 (1.14)	4.00 (1.11)	3.86 (0.99)	3.61 (0.95)	3.42 (0.95)

The background-luminance rating scores (i.e., the control condition) were also averaged for each participant. The mean and variance values are shown in Table 8. We conducted a two-way permutation analysis of variance for the background-luminance rating scores (Figure 4B). The main effect of the speed condition was significant (*F* = 2.94, *p* < 0.05, *r*^2^ < 0.01) while that of the delay condition was not significant (*F* = 0.05, *p*> 1.000, *r*^2^ < 0.01). The interaction between them was not significant (*F* = 0.09, *p*> 1.000, *r*^2^ < 0.01).

**Table 8:** Average (standard deviation) of background-luminance rating scores.

	**Delay = 0 [ms]**	**250 [ms]**	**500 [ms]**	**750 [ms]**	**1,000 [ms]**
Speed = slow	2.06 (1.15)	2.01 (1.18)	2.04 (1.19)	2.04 (1.19)	2.04 (1.21)
Speed = medium	1.95 (1.22)	1.93 (1.16)	1.94 (1.17)	1.97 (1.16)	1.97 (1.14)
Speed = fast	1.92 (1.18)	1.91 (1.20)	1.90 (1.20)	1.92 (1.19)	1.92 (1.22)

SoA rating scores decreased as the delay increased, which was a successful replication of previous studies showing that the delay between an action and its effect is an important factor for causal perception, i.e., sense of agency (e.g., Sato and Yasuda, 2005; Ebert and Wegner, 2010; Kalckert and Ehrsson, 2012; Kawabe, 2013; Kawabe et al., 2013; Rognini et al., 2013; Wen et al., 2015b). Since the background-luminance rating scores were not affected by the delay, the results relating to SoA were unlikely to originate from unintended factors independent of the targeted sensation of the judgement (e.g., response bias and/or demand characteristics).

SoA rating scores decreased as the speed of the temporal luminance change decreased. The results are consistent with the previous study (Kawabe, 2013) showing that sense of agency increased with the magnitude of a participant's action feedback. On the other hand, there is no *a priori* reason to assume that the speed of visual feedback influences the sense of agency. One possibility suggested by a previous study (Kawabe, 2013) is that the larger magnitude of a participant's action feedback is perceived as an indication of the larger influence of the action on the external world. Hence, a greater speed of visual feedback might entail a stronger sense of agency. Another possibility is that the effect of the speed of the temporal luminance change on the sense of agency came from the visual interpretation of the feedback. As described above, slower motion is a diagnostic feature for heavier and/or stiffer objects (Todd and Warren, 1982; Shim et al., 2009; Kawabe et al., 2015; Kawabe and Nishida, 2016; Bi et al., 2018). In general, heavier and/or stiffer objects cannot be well controlled. As such, the visual interpretation of the feedback may determine the SoA rating. Another possibility is that the total trial time until the end of the luminance change, rather than the speed itself, might have affected the SoA evaluation. Participants might feel a loss of agency when their effort in holding down the key was not as effective as they wished under the assumption that they wanted to complete each trial as quickly as possible.

The background-luminance rating scores also increased as the speed of the temporal luminance change increased. However, the range of the rating score variation was smaller for the background luminance change than for the sense of agency. In comparison with the scores in the catch trials, in which actual background luminance change occurred, the scores in the experimental trials were fairly low. Hence, we concluded that the effect of the speed of the temporal luminance change on the perception of background luminance change was small because it might be explained by a response bias or the misattribution of slow luminance change to its background.

**Figure 5:**
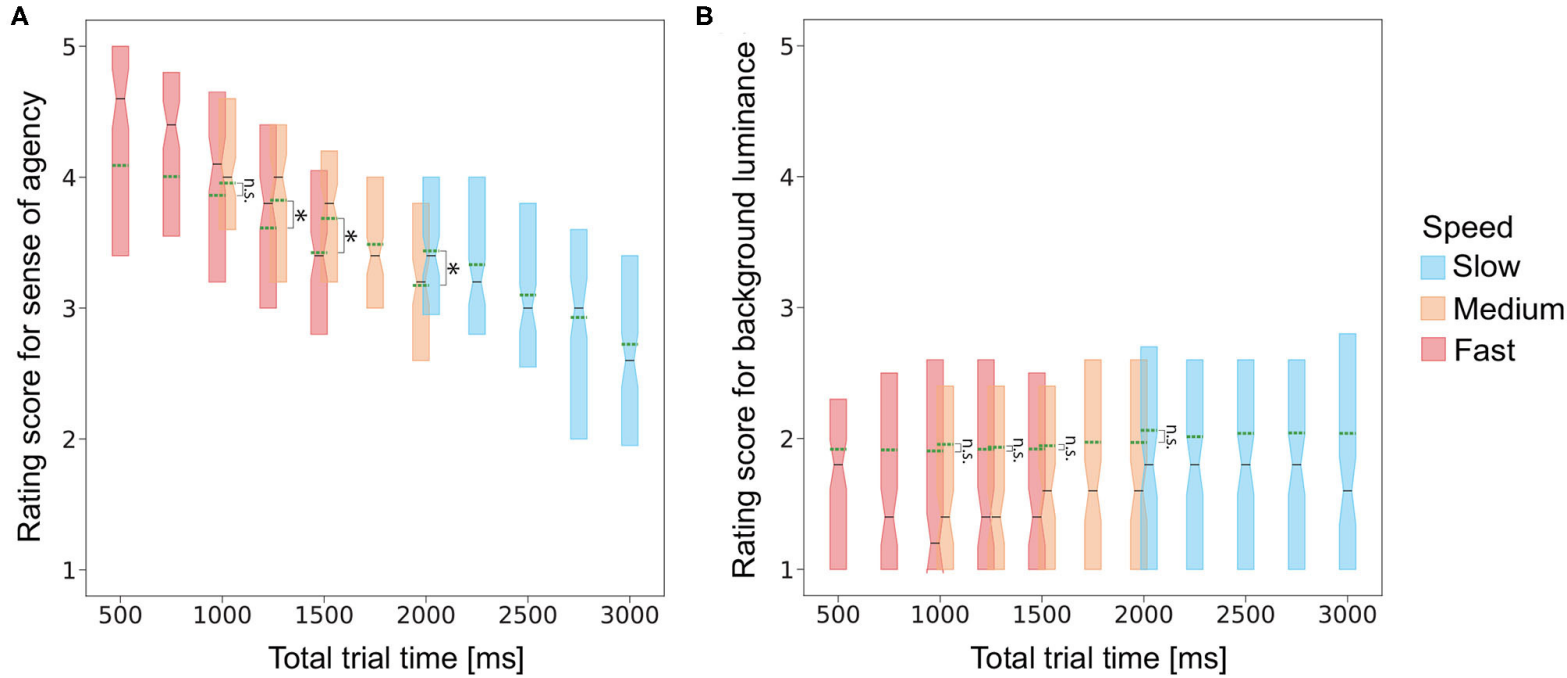
Box plot of the **(A)** SoA and **(B)** background luminance rating scores as a function of total trial time. Green dashed lines denote the averaged values of the rating scores. The Asterisks and n.s. denotes that the difference was significant (i.e., Bonferroni corrected p <0.05) and not significant (i.e., Bonferroni corrected p > 0.05), respectively.

As in Experiment 1, we analyzed whether the single parameter, total trial time, can fully explain the rating scores for the SoA and background luminance. Plots of the rating scores for SoA and background-luminance as a function of total trial time are shown in Figure 5. Regression analyses of rating scores using speed, delay, combinations of speed and delay, and total trial time showed that total trial time could explain a large part of the rating scores (Table 6). In the detailed analysis, we found that the averaged SoA rating score under the condition (duration = 1,000 ms and delay = 250 ms) was significantly higher than that under the condition (duration = 500 ms and delay = 750 ms) (Cliff's delta = 0.11, Bonferroni corrected *p* < 0.001), that the averaged SoA rating score under the condition (duration = 1,000 ms and delay = 500 ms) was significantly higher than that under the condition (duration = 500 ms and delay = 1,000 ms) (Cliff's delta = 0.16, Bonferroni corrected *p* < 0.001), and that the averaged SoA rating score under the condition (duration = 2,000 ms and delay = 0 ms) was significantly higher than that under the condition (duration = 1,000 ms and delay = 1,000 ms) (Cliff's delta = 0.18, Bonferroni corrected *p* < 0.001). We did not observe a significant difference for the combinations of 1,000 ms. These results suggest that the total trial time is possibly a major parameter for explaining the SoA rating scores, while the two independent parameters, speed and delay, also somehow impact independently on the scores. Upon the major role of the total trial time, it is probably natural to interpret the effect of speed as the effect of stimulus duration. To further disentangle the contributions of the total trial time, speed and stimulus duration, it is necessary to test conditions wherein these parameters are varied independently in future studies. Unlike the results for SoA, the average scores for background luminance were not significantly different between all pairs.

**Figure 6:**
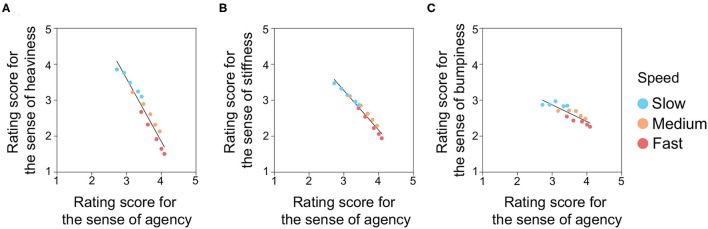
Scatter plots of the rating scores for **(A)** heaviness, **(B)** stiffness, and **(C)** bumpiness as a function of the rating scores for the sense of agency. The black lines denote fitted lines by simple regression analysis. In each panel, 15 conditions (3 speeds × 5 delays) are plotted. All *p*-values were Bonferroni-corrected.

As mentioned above, we expected that the SoA rating scores would be influenced by the delay and speed in the opposite direction to the rating scores for heaviness and stiffness obtained in Experiment 1. To confirm this expectation, using 15 experimental conditions (5 delays × 3 speeds), we conducted a linear regression analysis of the rating scores for heaviness and stiffness by treating the SoA scores as an independent variable (Figure 6). The results showed that the slopes were significantly below zero in the rating scores for heaviness (slope = −1.75, *t*(13) = −13.66, Bonferroni-corrected *p* < 0.001, *R*^2^ = 0.93), stiffness (slope = −1.10, *t*(13) = −19.17, Bonferroni-corrected *p* < 0.001, *R*^2^ = 0.97), and bumpiness (*slope* = −0.47, *t*(13) = −5.92, Bonferroni-corrected *p* < 0.001, *R*^2^ = 0.73). It is worth noting that both the coefficient of determination and the absolute slope value of the fitted function are larger for heaviness and stiffness than those for bumpiness. This is possibly because the perception of heaviness and stiffness is influenced by the onset delay of temporal luminance changes, which is related to sensorimotor congruence, while the perception of bumpiness is not. These results suggest that the brain uses the delay and speed of the visual feedback to determine the sense of agency in a way similar to the estimation of heaviness and stiffness.

## 4. General Discussion

We found that the heaviness/stiffness evaluation for the temporal luminance changes as visual feedback of a participant's keystroke depended strongly on the delay and speed of the visual feedback. The bumpiness and background-luminance change evaluations were not influenced by the delay of the visual feedback. Similarly to the heaviness/stiffness evaluations, the sense of agency evaluations was influenced by both the delay and speed of the visual feedback. A simple regression analysis showed that the delay and speed influenced heaviness and stiffness evaluations in the opposite direction to the sense of agency rating.

The present study offers the first evidence that the delay and speed of the visual feedback of a participant's keystroke are important parameters for the illusory perception of heaviness/stiffness. Although some previous studies in engineering contexts have shown that the change in color of a hand-shaped mouse cursor or a participant's finger when pressing a button or a real object enhanced the stiffness sensation (Argelaguet et al., 2013; Punpongsanon et al., 2015), they did not investigate the role of the delay and speed of the visual feedback in the determination of illusory heaviness/stiffness. The present study independently manipulated the delay and speed of the visual feedback and found that both parameters could strongly and interactively contribute to illusory heaviness/stiffness. Appropriately extending the parameters of the visual feedback which the present study has revealed, in the future, it may be possible to finely control the heaviness and stiffness sensation.

As expected, we observed that the delay and speed influenced the rating scores for the sense of agency and the heaviness/stiffness in the opposite direction. The results indicate that the sensorimotor incongruence between the timing of a participant's keystroke and that of visual feedback is a key determinant of both the sense of agency and the illusory heaviness/stiffness. It remains an open question whether the opposite tendency between the sense of agency and the illusory heaviness/stiffness persists when the range of the delay and speed of visual feedback is extended. For example, if the delay became greater than 1,000 ms, it is likely that the sense of agency would be completely lost while the perceived heaviness/stiffness would start to decrease since the temporal luminance changes would no longer be attributable to a participant's own keystroke. It is important to test this prediction in future studies. Another interesting direction would be to investigate how the sensorimotor incongruence in terms of spatial offset between a participant's hand and the feedback it triggers could influence the illusory heaviness/stiffness since the sense of agency is influenced by the spatial congruence between an action and the feedback it triggers (Farrer et al., 2008; Kalckert and Ehrsson, 2012).

One might suspect the possibility that the delay and speed of temporal luminance changes influenced the way the participants pressed an assigned key. For example, participants might physically apply a greater force to a key when the temporal luminance changes were presented with a larger delay and/or a lower speed, and the participants may interpret the visual feedback as a result of making the keystroke with a stronger physical force, as that would imply an object/event with a stronger heaviness/stiffness. Thus, we could conduct laboratory experiments to measure pressing forces while changing the delay and speed of the temporal luminance changes. On the other hand, even if we measured the force data during our task, it would be difficult to disentangle the following two possibilities: one possibility would be that the force change came directly from the delay and speed of the temporal luminance changes and the other possibility would be that the force change came from the illusory heaviness/stiffness. For the former possibility, it is not easy to theoretically justify why the delay and speed of visual feedback would cause a greater force to be applied to a key. For the latter possibility, the force change is the byproduct of the illusory heaviness/stiffness, and hence, the force itself cannot underlie the illusory heaviness/stiffness. Therefore, at this stage, we believe that there is not a plausible reason to investigate the effect of the force applied to a key during our task on illusory heaviness/stiffness. Still, it is necessary to empirically specify the role of force applied to a key in the determination of the illusory heaviness/stiffness in future studies.

Several levels of potential mechanisms may be able to explain our results. First, the results may be explained by a model assuming a low-level mechanism in which the degree of visual-motor incongruence modulates the perceived weight of the object actually being grasped (Honda et al., 2013). In our experiment, since we did not instruct participants to judge such haptic weight and since they did not actually grasp an object, we cannot directly apply the explanation of the earlier study to our results.

The earlier study (Honda et al., 2013) also proposed a model in which participants constantly updated their predictions of visual outcomes for the estimation of weight. If this model were to be applied to the results in our experiment, the prediction of the delay and speed parameters would end up converging as intermediate values since the participants could learn the range of the delay and speed parameters during the experiment. If this were the case, the heaviness and stiffness sensations should have been at their minimum when the intermediate delay and speed condition was presented. However, in our results, the minimum values for heaviness and stiffness were observed when the delay was 0 ms and the speed was fast. Therefore, rather than a short-term updating of the temporal relationship between the participants self-action and the feedback provided in response to it, it is plausible to assume that the participants were always able to predict the situation with a delay of 0ms and a fast speed. The reason why the heaviness and stiffness evaluations were minimized under the 0 ms delay condition is probably because the evaluations were made based on the evolutionarily cultivated situation where there is no visual feedback delay in the perceptual timing of an action. In addition, the reason why the evaluations were minimized under the fast speed condition might be because the evaluation was based on the statistical association that lighter objects can be moved faster (rather than on prediction error). These models are not likely to be updated unless the participants are exposed to the same stimuli for a long time, as in the case of adaptation.

Another study (Takamuku and Gomi, 2015) argued that the brain re-selects our internal model for specific physical events on the basis of motion information in the visual feedback when detecting sensorimotor incongruence, rather than that the brain directly uses the sensorimotor incongruence to calculate the force. In their experiments, a cursor was shown to the participants as visual feedback, and in some trials a delay was added to the display of the cursor. Based on the non-arbitrary relationship between one's action and the feedback it triggers, the brain might be able to easily re-select the internal model on the basis of the sensorimotor incongruence. The present study differs from these earlier studies in that the causal relationship between a participant's keystroke and the feedback it triggers was arbitrary. In other words, the outcome of action in the present study was a temporal luminance change that could stem from various physical sources and was not specifically linked with the keystroke. For such an arbitrary action-feedback relationship, it is unclear whether the explanation of a re-selection of the internal model is straightforwardly applicable to the illusory heaviness/stiffness perceived during a participants' keystroke in the present study. We speculate that rather than the re-selection of the internal model for physical events, the internal model for a general statistical relationship between the weight (or stiffness) of an object and the delay of its movement after the application of force to it possibly produces the illusory heaviness/stiffness for the arbitrary action-feedback causal relationship. For example, after the application of force to an object, a heavier (or stiffer) object moves (or deforms) more slowly than a lighter (or less stiff) one, and thus shows more delay in moving (or deforming) to a certain position (or shape). The brain may take advantage of the statistical relationship between the timing and speed of an action's consequence in order to judge the heaviness and stiffness of an object. To confirm this speculation, future studies need to assess how the degree of arbitrariness between an action and its feedback influences the strength of the illusory heaviness/stiffness.

Some higher-level mechanisms might also explain the results in this study. The first candidate mechanism is expectation about the total trial time. In the task, the participants were exposed to stimuli having various temporal lengths, and thus, they likely established some expectations about the total trial time. Such expectations might have affected their judgment of the heaviness/stiffness sensations. The second candidate mechanism is the avoidance of error punishments. In the task, the participants were required to keep pressing the key longer as the temporal length of the stimuli increased. Thus, for the longer stimuli, the possibility for the participants to be punished for erroneous key releases increased. The greater inclination toward punishment avoidance with the longer stimuli might have caused greater heaviness/stiffness rating scores. There is also a possibility that the punishment avoidance might have invoked some negative emotion, which possibly influenced the judgment. The final candidate mechanism is that of attentional demand. Specifically, the simple maintenance of the participants attention toward the task might have felt more demanding in the longer than the shorter tasks, and the different levels of this attentional demand might have influenced the heaviness/stiffness judgments. Consistent with these interpretations based on the total trial time, additional analysis showed that total trial time could explain a large part of the ratings scores, although not completely. Also, the fact that the ratings scores did not saturate even after a delay of 1,000 ms may be interpreted as a result of higher-order cognitive functions rather than lower-level sensorimotor incongruence. In future research, it would be important to conduct experiments excluding higher-level effects such as expectation and to separate them from lower-level effects.

We provided our participants with the written instruction “Evaluate the sense of heaviness youfelt while controlling the square's luminance change” without emphasizing the haptic (e.g., muscle effort and key resistance) or visual (e.g., square seems heavy) aspects of the heaviness/stiffness sensation. This was because we believe that it was not so easy to ascribe the sensation to haptics or vision, and moreover, that the sensation was possibly the product of the bi-modal processing or cross-modal integration between the participants self-action and the visual feedback provided in response to it. On the other hand, there is room to improve the experimental design by including instructions that clarify the underlying mechanism for the sensation. For example, it would be possible to assess the extent to which haptic and/or vision involves the heaviness/stiffness sensation by asking the participants to haptically evaluate the sensation using actual weight stimuli or by asking them to visually evaluate the sensation without making any keystrokes.

## Data Availability Statement

The original contributions presented in the study are included in the article/Supplementary Material, further inquiries can be directed to the corresponding author.

## Ethics Statement

The studies involving human participants were reviewed and approved by the Ethics committee of Nippon Telegraph and Telephone Corporation. The patients/participants provided their written informed consent to participate in this study.

## Author Contributions

TY and TK designed the experiments and wrote the paper. TY collected and analyzed the data. Both authors contributed to the article and approved the submitted version.

## Conflict of Interest

TY and TK are employees of NTT Communication Science Laboratories, which is the basic-science research section of Nippon Telegraph and Telecommunication corporation NTT. There is a pending patent involving the reported research. There are no products in development or marketed products to declare. The pending patent does not alter the authors' adherence to the policies of the Frontiers in neuroscience.

## Publisher's Note

All claims expressed in this article are solely those of the authors and do not necessarily represent those of their affiliated organizations, or those of the publisher, the editors and the reviewers. Any product that may be evaluated in this article, or claim that may be made by its manufacturer, is not guaranteed or endorsed by the publisher.
