# Supplementary material for: Delay and Speed of Visual Feedback of a Keystroke Cause Illusory Heaviness and Stiffness
Source: Front Neurosci. 2022 Mar 18;16:761697. doi: 10.3389/fnins.2022.761697 (PMC8972167; doi:10.3389/fnins.2022.761697)
Supplement: Supplementary file 1 [file Presentation_1.PDF]

Supplemental Material

Delay and speed of visual feedback of a keystroke cause illusory heaviness and stiffness

Takumi Yokosaka* and Takahiro Kawabe

*Corresponding author: yokosaka.takumi@gmail.com

S1. Instructions for participants

The instructions presented to participants in this study were written in Japanese. In this section, we show the original instruction images and provide a translation.

S1.1 Experiment 1: heaviness evaluation task

We showed the instruction image shown in Fig. S1 to participants before the experiment began. In the following paragraph, we show English translations of the Japanese text in this image.

- The title reads, "Contents of Experiment."
- The first paragraph in this image reads, "From now on, a white or black square will be displayed in the center of your monitor."
- The second paragraph in this image reads, "Pressing the 'M' key on your keyboard can change the luminance of the square. **Hold the 'M' key down until the screen has changed.** (If you release the key before the screen finishes changing, the screen will return to its initial state.)"
- The third paragraph in this image reads, "After the screen has changed, please evaluate **the sense of heaviness you felt while controlling the square's luminance change** using a five-point scale by pressing one of the following number keys."
- The sentence to the left of the black arrow reads, "**Felt no heaviness sensation**".
- The sentence to the right of the black arrow reads, "**Felt a strong heaviness sensation**".
- The last paragraph in this image reads, "This experiment will end when you have completed all of the prescribed trials. Please end the experiment by pressing the 'space key'."

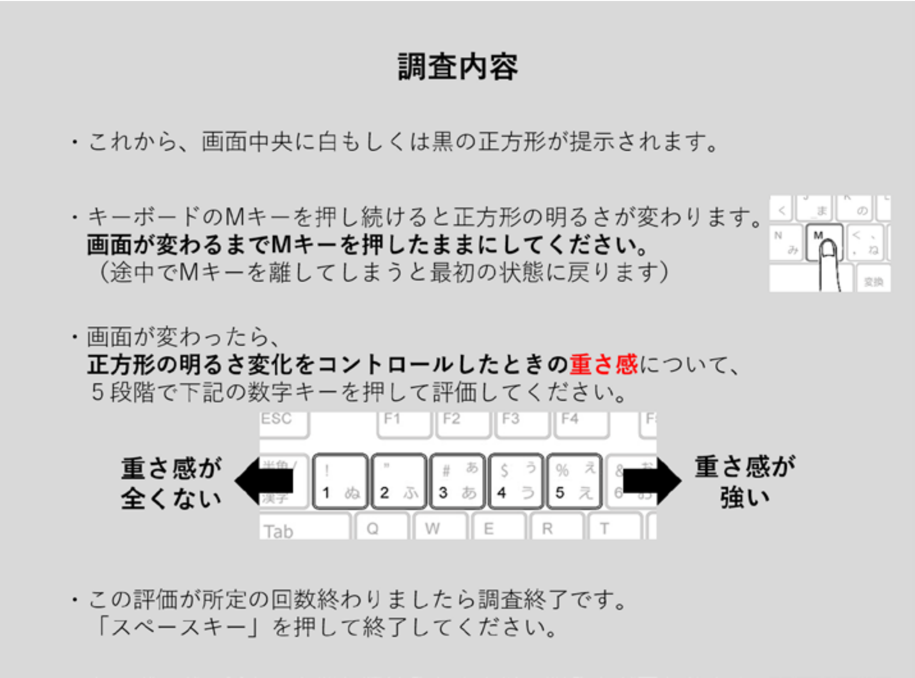

Figure S1. Instruction image presented before the heaviness evaluation task.

On the answer screen, the instruction image shown in Fig. S2 was displayed. The text above the illustration of the keyboard reads, "Please evaluate **the sense of heaviness** **you felt while controlling the square's luminance change** using a five-point scale by pressing one of the following number keys." The sentence to the left of the black arrow reads, "**Felt no heaviness sensation**". The sentence to the right of the black arrow reads, "**Felt a strong heaviness sensation**".

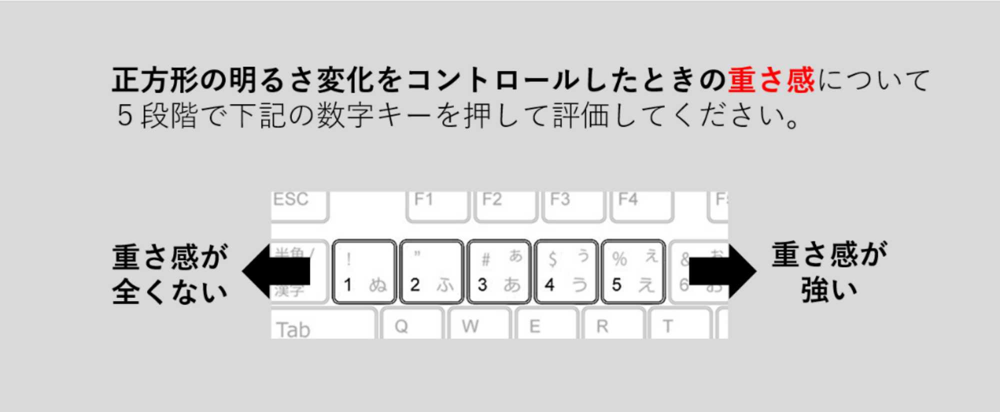

Figure S2. Instruction image presented on the answer screen of the heaviness evaluation task.

S1.2 Experiment 1: stiffness evaluation task

We showed the instruction image shown in Fig. S3 to participants before the experiment began. In the following paragraph, we present English translations of the Japanese text in this image.

- The title reads, "Contents of Experiment."
- The first paragraph in this image reads, "From now on, a white or black square will be displayed in the center of your monitor."
- The second paragraph in this image reads, "Pressing the 'M' key on your keyboard can change the luminance of the square. **Hold the 'M' key down until the screen has changed.** (If you release the key before the screen finishes changing, the screen will return to its initial state.)"
- The third paragraph in this image reads, "After the screen has changed, please evaluate **the sense of stiffness you felt while controlling the square's luminance change** using a five-point scale by pressing one of the following number keys."
- The sentence to the left of the black arrow reads, "**Felt no stiffness sensation**".
- The sentence to the right of the black arrow reads, "**Felt a strong stiffness sensation**".
- The last paragraph in this image reads, "This experiment will end when you complete all of the prescribed trials. Please end the experiment by pressing the 'space key'."

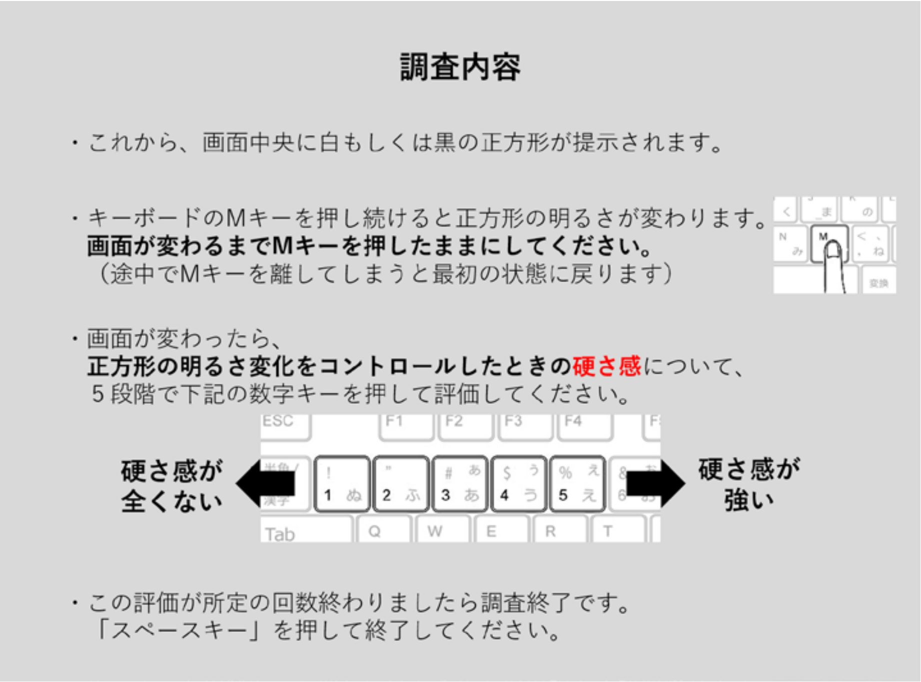

Figure S3. Instruction image presented before the stiffness evaluation task.

On the answer screen, the instruction image shown in Fig. S4 was displayed. The text above the illustration of the keyboard reads, "Please evaluate **the sense of stiffness** **you felt while controlling the square's luminance change** using a five-point scale by pressing one of the following number keys." The sentence to the left of the black arrow reads, "**Felt no stiffness sensation**". The sentence to the right of the black arrow reads, "**Felt a strong stiffness sensation**".

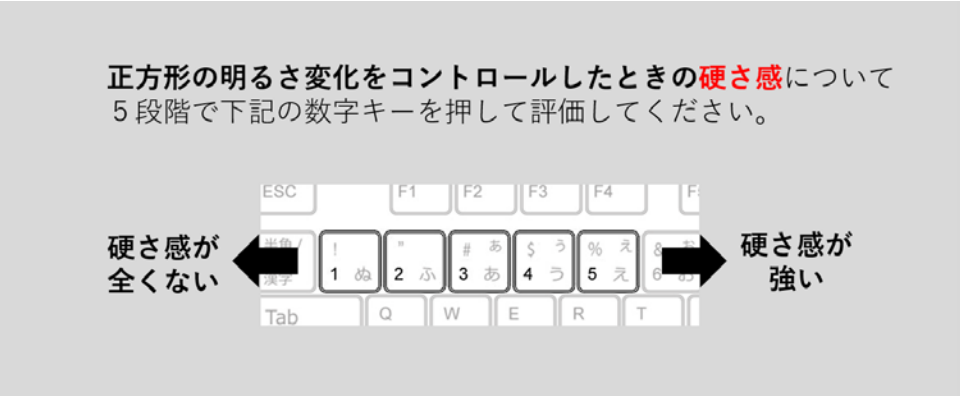

Figure S4. Instruction image presented on the answer screen of the stiffness evaluation task.

S1.3 Experiment 1: bumpiness evaluation task

We showed the instruction image shown in Fig. S5 to participants before the experiment began. In the following paragraph, we show English translations of the Japanese text in this image.

- The title reads, "Contents of Experiment."
- The first paragraph in this image reads, "From now on, a white or black square will be displayed in the center of your monitor."
- The second paragraph in this image reads, "Pressing the 'M' key on your keyboard can change the luminance of the square. **Hold the 'M' key down until the screen has changed.** (If you release the key before the screen finishes changing, the screen will return to its initial state.)"
- The third paragraph in this image reads, "After the screen has changed, please evaluate **the sense of bumpiness you felt while controlling the square's luminance change** using a five-point scale by pressing one of the following number keys."
- The sentence to the left of the black arrow reads, "**Felt no bumpiness sensation**".
- The sentence to the right of the black arrow reads, "**Felt a strong bumpiness sensation**".
- The last paragraph in this image reads, "This experiment will end when you complete all of the prescribed trials. Please end the experiment by pressing the 'space key'."

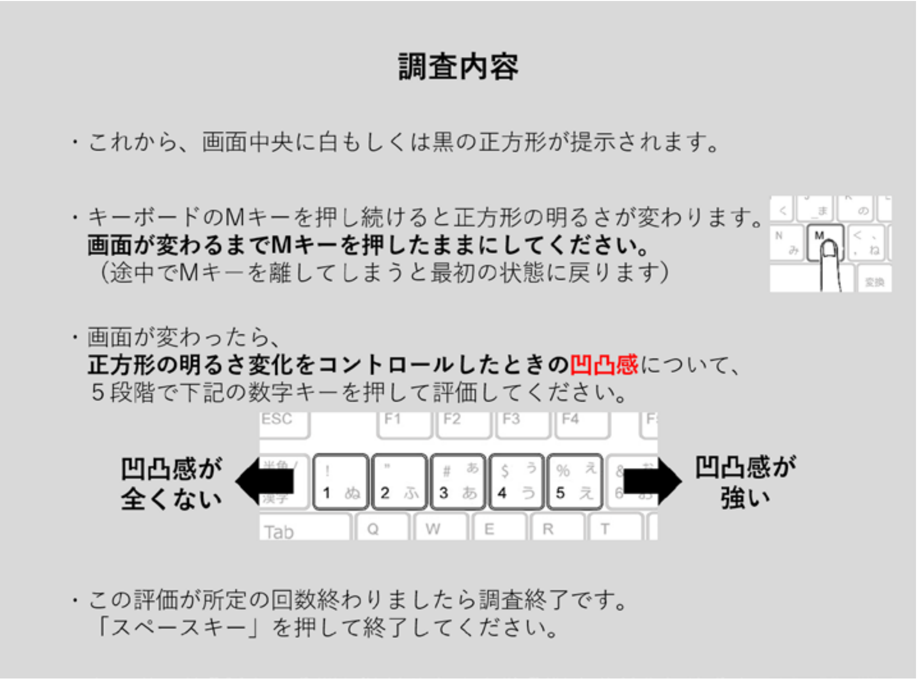

Figure S5. Instruction image presented before the bumpiness evaluation task.

On the answer screen, the instruction image shown in Fig. S6 was displayed. The text above the illustration of the keyboard reads, "Please evaluate **the sense of bumpiness** **you felt while controlling the square's luminance change** using a five-point scale by pressing one of the following number keys." The sentence to the left of the black arrow reads, "**Felt no bumpiness sensation**". The sentence to the right of the black arrow reads, "**Felt a strong bumpiness sensation**".

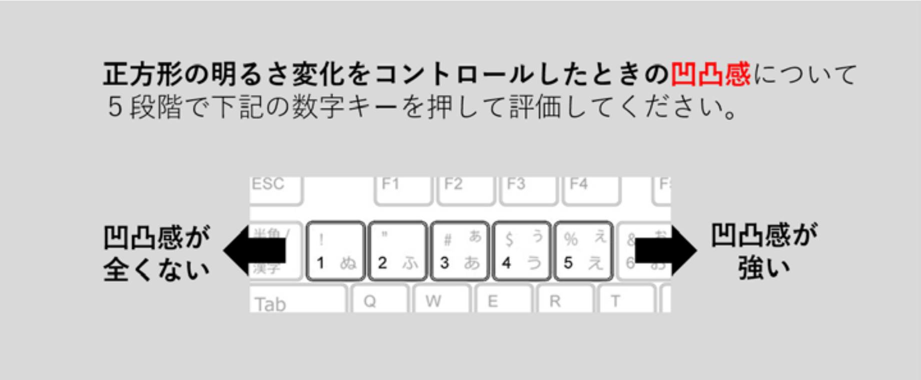

Figure S6. Instruction image presented on the answer screen of the bumpiness evaluation task.

S1.4 Experiment 2: SoA evaluation task

We showed the instruction image shown in Fig. S7 to participants before the experiment began. In the following paragraph, we show English translations of the Japanese text in this image.

- The title reads, "Contents of Experiment."
- The first paragraph in this image reads, "From now on, a white or black square will be displayed in the center of your monitor."
- The second paragraph in this image reads, "Pressing the 'M' key on your keyboards can change the luminance of the square. **Hold the 'M' key down until the screen has changed.** (If you release the key before the screen finishes changing, the screen will return to its initial state.)"
- The third paragraph in this image reads, "After the screen has changed, please evaluate **the ease with which you were able to change the luminance of the square** **by pressing and holding the 'M' key** using a five-point scale by pressing one of the following number keys."
- The sentence to the left of the black arrow reads, "**It was very difficult to change the luminance**".
- The sentence to the right of the black arrow reads, "**It was very easy to change the luminance**".
- The last paragraph in this image reads, "This experiment will end when you complete all of the prescribed trials. Please end the experiment by pressing the 'space key'."

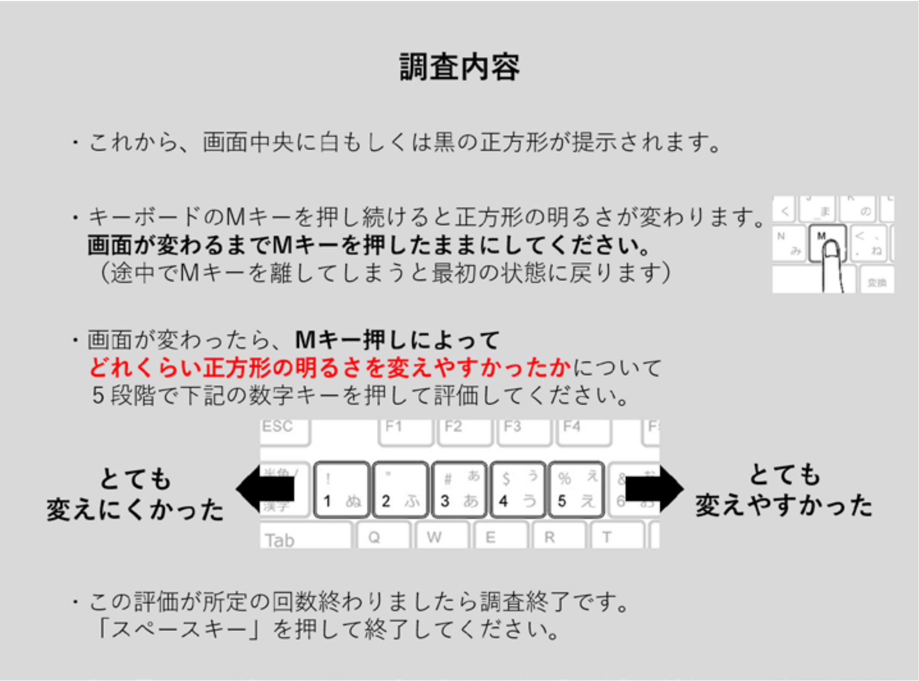

Figure S7. Instruction image presented before the SoA evaluation task.

On the answer screen, the instruction image shown in Fig. S8 was displayed. The text above the illustration of the keyboard reads, "Please evaluate **the ease with which you were able to change the luminance of the square by pressing and holding the ‘M’ key** using a five-point scale by pressing one of the following number keys." The sentence to the left of the black arrow reads, " **It was very difficult to change the luminance** ". The sentence to the right of the black arrow reads, " **It was very easy to change the luminance** ".

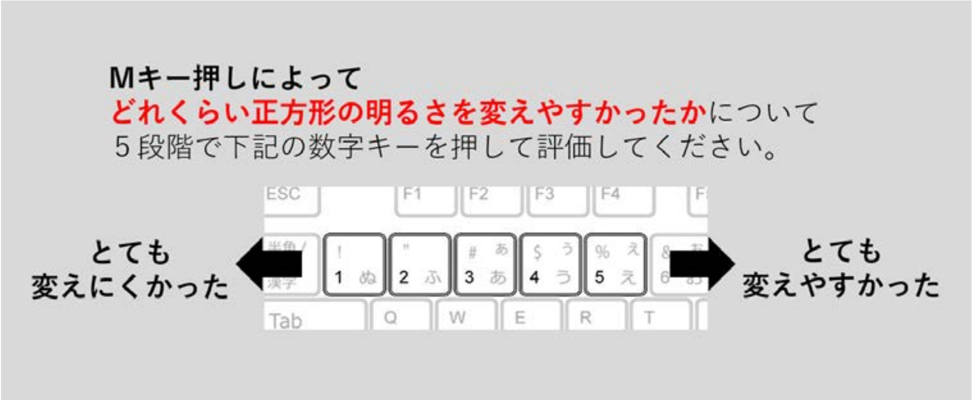

Figure S8. Instruction image presented on the answer screen of the SoA evaluation task.

S1.5 Experiment 2: background-luminance evaluation task

We showed the instruction image shown in Fig. S9 to participants before the experiment began. In the following paragraph, we show English translations of the Japanese text in this image.

- The title reads, "Contents of Experiment."
- The first paragraph in this image reads, "From now on, a white or black square will be displayed in the center of your monitor."
- The second paragraph in this image reads, "Pressing the 'M' key on your keyboard can change the luminance of the square. **Hold the 'M' key down until the screen has changed.** (If you release the key before the screen finishes changing, the screen will return to its initial state.)"
- The third paragraph in this image reads, "After the screen has changed, please evaluate **the degree of the change in the screen's background luminance (i.e., not the square's luminance) felt while pressing and holding the 'M' key** using a five-point scale by pressing one of the following number keys."
- The sentence to the left of the black arrow reads, "**The background luminance did not change at all.**"
- The sentence to the right of the black arrow reads, "**The background luminance changed greatly.**"
- The last paragraph in this image reads, "This experiment will end when you complete all of the prescribed trials. Please end the experiment by pressing the 'space key'."

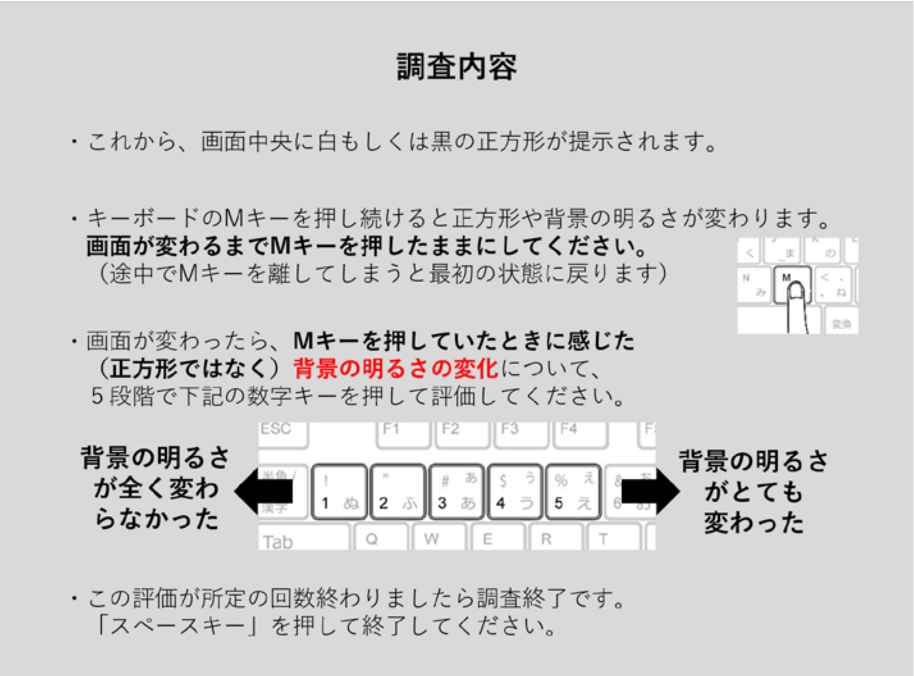

Figure S9. Instruction image presented before the background-luminance evaluation task.

On the answer screen, the instruction image shown in Fig. S10 was displayed. The text above the illustration of the keyboard reads, "Please evaluate **the degree of the change in the screen's background luminance** **(i.e., not the square's luminance) felt while pressing and holding the 'M' key** using a five-point scale by pressing one of the following number keys." The sentence to the left of the black arrow reads, " **The background luminance did not change at all.**" The sentence to the right of the black arrow reads, " **The background luminance changed greatly.**"

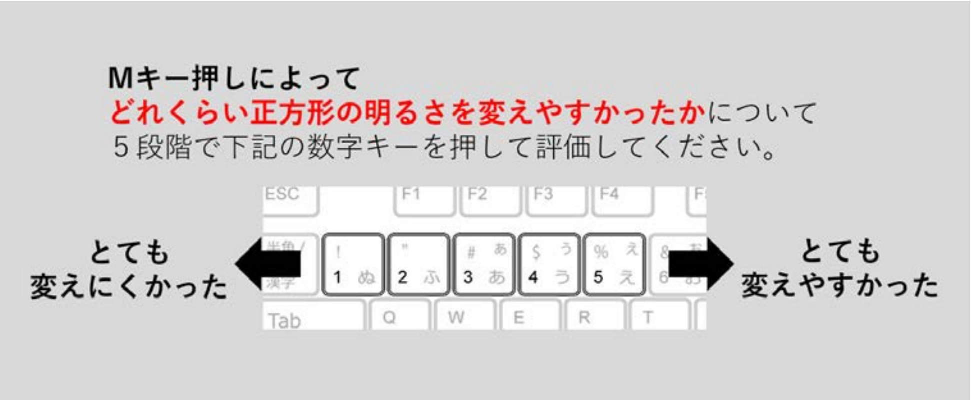

Figure S10. Instruction image presented on the answer screen of the background-luminance evaluation task.

S2. Results of Preliminary measurements of latency

We made preliminary measurements of rough latency, i.e., how long it took for the luminance of the square to change after pressing a key, by attaching a pressure sensor to the keyboard and a CdS cell to the monitor of the author's computer. From the results of three measurements under the 0 ms delay condition, we found that the latency ranged from a minimum of 38 milliseconds to a maximum of 106 milliseconds (Figure S11A). The latency likely stemmed from system delay and the mean $\pm$ SD of the latency for the three measurements was 76.12 $\pm$ 8.02 ms. Moreover, the averaged SD of overall duration (i.e., from the time of the key press to the time the luminance of the square had finished changing) was 17.86 ms, which is much shorter than the minimum difference of 250 ms between the delay conditions, and did not depend on the delay and speed conditions (see Figure S11B).

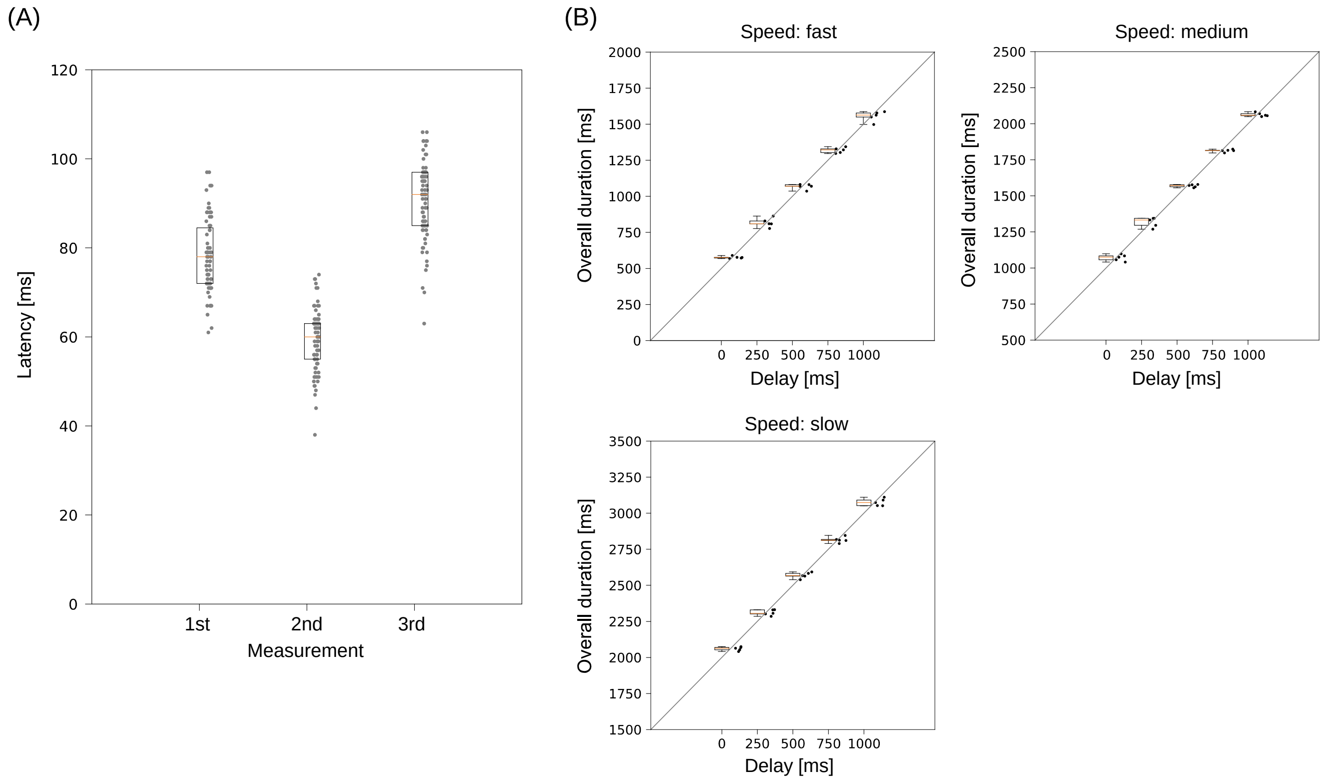

Figure S11. (A) Preliminary measurements of latency, which is defined as the temporal separation between the key press and the onset of luminance change under a 0 ms delay condition on the author’s PC. (B) Preliminary measurements of overall duration (i.e., latency + delay + duration) on the author's PC.

S3. Analysis of effective frames per second (efps)

Figs. S12A and S12B show means and standard deviations of efps for each participant in each experiment. To confirm that participants with a mean efps of 30 Hz or less did not exhibit a larger standard deviation of efps, we showed a scatter plot of the mean and standard deviation of efps for each participant in Fig. S12C. The results showed that most participants' PCs performed the experiments at almost 60 efps with small fluctuations, which suggests that it is unlikely that the rendering performance of participants' PCs differed significantly between trials.

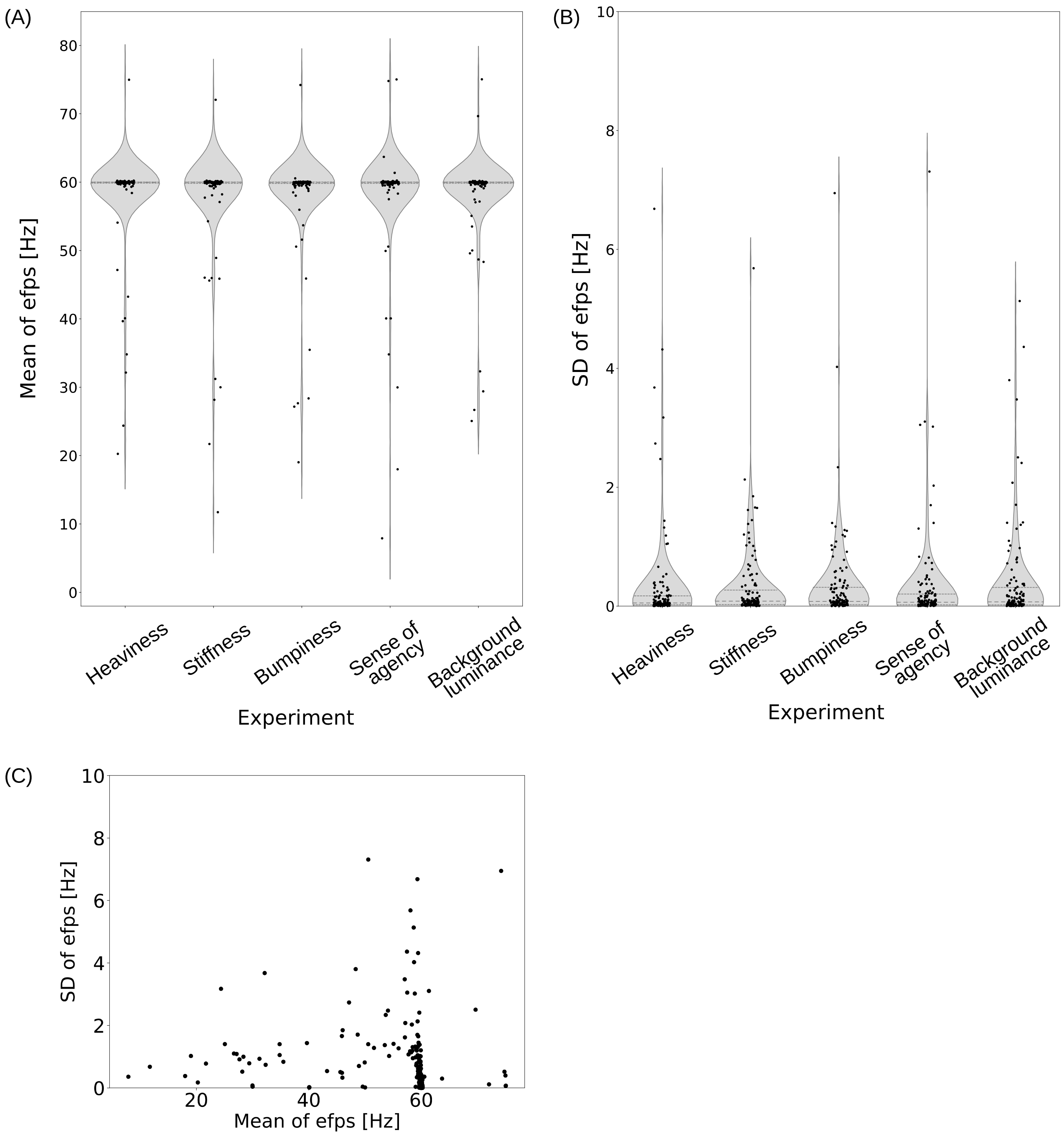

Figure S12. (A) Means of efps across participants. (B) Standard deviations of efps across participants. (C) Scatter plots of means and standard deviations of efps. Each black circle denotes mean and/or standard deviation averaged across trials for each participant.
